# Periconceptual Caffeine Intake and Adverse Pregnancy Outcomes: Results From the nuMoM2b Cohort

**DOI:** 10.1111/1471-0528.70018

**Published:** 2025-09-23

**Authors:** Rachel S. Ruderman, Alexa A. Freedman, Sunitha C. Suresh

**Affiliations:** ^1^ University of Chicago/Endeavor Health Chicago Illinois USA; ^2^ Northwestern University/Endeavor Health Evanston Illinois USA; ^3^ University of Chicago/Endeavor Health Evanston Illinois USA

**Keywords:** caffeine, counselling, nutrition, periconception, pregnancy, risks

## Abstract

**Objective:**

To determine the association of self‐assessed caffeine consumption with adverse pregnancy outcomes (APO).

**Design:**

Secondary analysis of births in the Nulliparous Pregnancy Outcomes Study: Monitoring Mothers‐to‐be (nuMoM2b). Caffeine intake was assessed by the self‐reported Food Frequency Questionnaire reflecting consumption in the three months prior to the first trimester visit.

**Setting:**

nuMoM2b was a prospectively collected cohort.

**Population:**

This is a large US‐based cohort of pregnant patients.

**Methods:**

High caffeine intake was defined as ≥ 200 mg/day. Logistic models assessed associations between high intake and APO, adjusted for confounders. We also grouped caffeine intake in 50 mg increments and tested whether increased consumption was associated with increased odds of APO.

**Main Outcome Measures:**

APO were defined as a composite of intrauterine foetal demise > 20 weeks' gestation, hypertensive disorders of pregnancy, preterm birth and small for gestational age infant.

**Results:**

The primary analysis included 7345 participants with live births or pregnancy loss > 20 weeks' gestation, for whom the median daily caffeine intake was 63.28 mg/day. 841 (11.4%) of patients had high intake and 2168 (29.5%) had an APO. When adjusted for confounders, high intake was not associated with increased odds of APO (aOR 0.99, 95% CI 0.84–1.16), nor was it associated with any individual APO. We found no significant increased odds of APO per 50 mg increase in caffeine intake.

**Conclusions:**

High caffeine intake assessed in the periconceptual period was not associated with the risk of APO. Further research exploring biomarkers and longitudinal childhood outcomes is needed to clarify limitations with regard to intake.

## 1 Introduction

Most women continue to consume caffeine during pregnancy [1]. Caffeine crosses the blood‐placental barrier and the foetus does not have the enzymes required for its metabolism [1, 2]. The Royal College of Obstetricians and Gynaecologists recommends limiting intake to < 200 mg per day in patient‐facing materials [3]. The American College of Obstetricians and Gynecologists (ACOG) similarly states that moderate caffeine consumption (< 200 mg per day) does not appear to be a major factor in miscarriage or preterm birth, a statement which many providers have used to recommend no more than 200 mg of caffeine during pregnancy [4].

Limited prospective and randomised trials have provided conflicting results regarding the relationship between caffeine and adverse pregnancy outcomes, including preterm birth and foetal growth restriction [2, 4, 5]. Existing data is limited by a lack of control for tobacco and alcohol use, inability to accurately estimate caffeine consumption, and non‐uniformity in defining the primary outcome [6, 7].

A 2015 Cochrane review found that reducing caffeine intake (by an average of 182 mg per day) did not affect birthweight, preterm birth, or small for gestational age infants [8]. A more recent secondary analysis of the National Institute of Child Health and Human Development Fetal Growth Studies—Singleton Cohort, a large prospective study, found that women who consumed low to moderate levels of caffeinated beverages early in the second trimester had a lower risk of gestational diabetes (OR 0.53, 95% CI 0.35–0.80), and found no increased risk for preeclampsia among those who consumed > 200 mg/day (OR 2.74, 95% CI 0.56–13.30) [9]. Conclusions based on single cohorts, or on caffeine exposure in the second and third trimesters despite evidence suggesting the theoretical risk is greatest in the first trimester, complicate our interpretations of these findings [2, 7].

In 2021, James et al. published a large‐scale narrative review which concluded that the existing body of evidence from observational studies and meta‐analyses demonstrates an association with maternal caffeine use and major negative pregnancy outcomes; the authors therefore suggested complete avoidance of caffeine in pregnancy [7]. Given the inconsistencies in previously published findings and recommendations which impact the vast majority of pregnant patients, we sought to evaluate the association between periconceptual caffeine consumption and adverse pregnancy outcomes using data from the prospective Nulliparous Pregnancy Outcomes Study: Monitoring Mothers‐to‐Be (nuMoM2b) [10]. We additionally sought to explore the risk of pregnancy loss prior to 20 weeks' gestation as a secondary outcome and separate analysis given its relevance in counselling for both patients and providers.

## 2 Materials and Methods

### 2.1 Study Overview

This is a secondary analysis of nuMoM2b. Institutional Review Board approval and a Data Use Agreement were obtained before data analysis began. This study is one of the largest prospective population‐based cohorts of pregnant women in the United States, which explored maternal characteristics and environmental factors that predict adverse pregnancy outcomes [10]. The study enrolled 10 038 nulliparous pregnant singleton patients in their first trimester at 8 US medical centres from 2010 to 2013. Methods of the nuMoM2b dataset have been published previously [10]. Caffeine consumption was assessed using the modified Block 2005 Food Frequency Questionnaire [11] in English or Spanish at the first trimester study visit, completed between 6 and 14 weeks gestation, and reflected calculated caffeine consumption in the prior 3 months [12]. NutritionQuest used intake of coffee, tea and 40 other food items to calculate a total daily dose of caffeine (mg/day). Caffeine consumption was defined as low (0–less than 200 mg/day) or high (200 mg/day or greater) per recommendations [4, 13]. This study followed reporting requirements of the Strengthening the Reporting of Observational Studies in Epidemiology (STROBE) statement [14].

### 2.2 Outcomes

**FIGURE 1:**
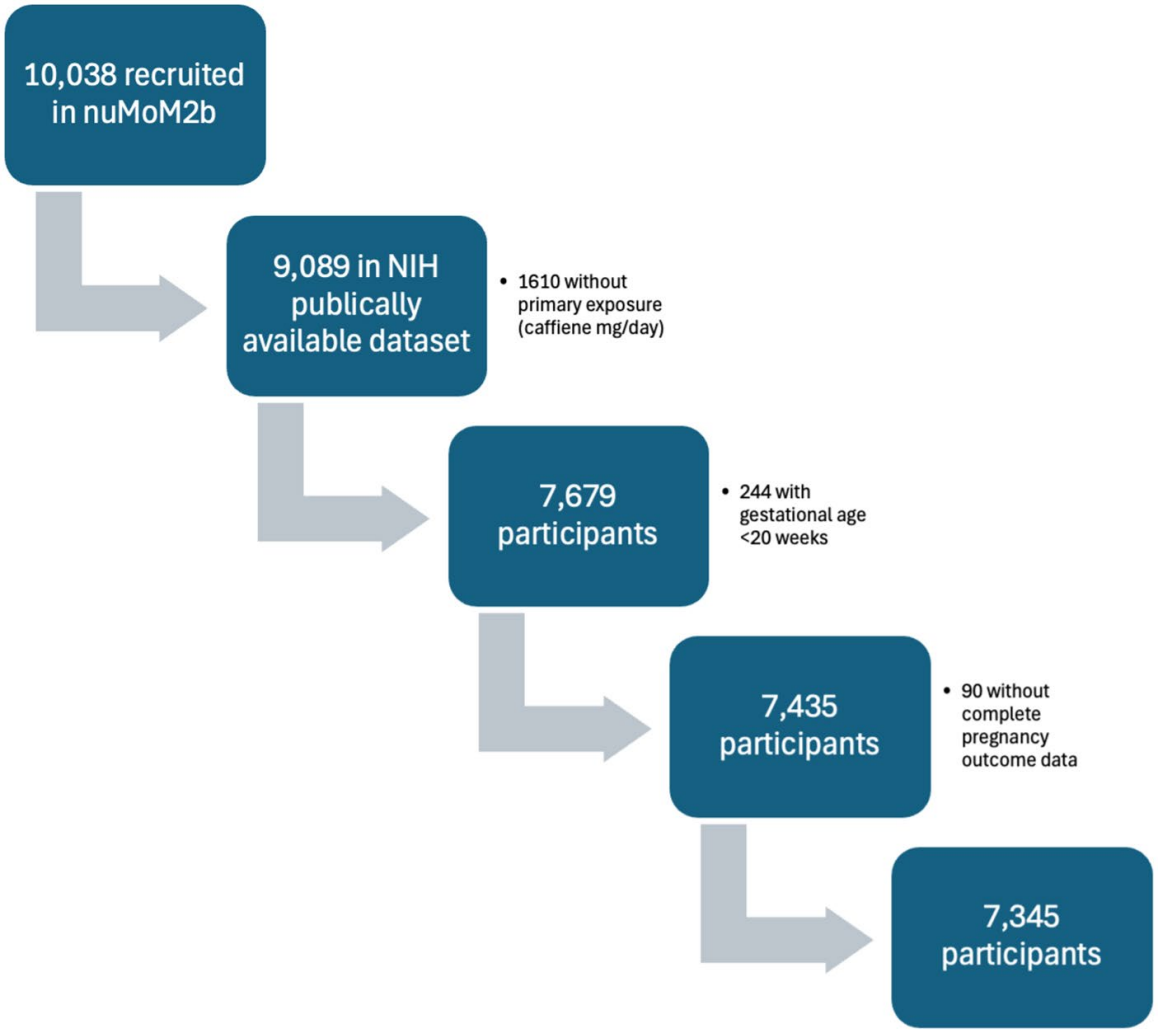
Flowchart of inclusion and exclusion criteria.

For our primary outcome, we included patients with complete caffeine data, patients who completed 20 weeks' gestational age of pregnancy, and excluded those without complete pregnancy outcome data (Figure 1). The primary outcome was defined as a composite of adverse pregnancy outcomes (APO), which included one or more of the following: stillbirth (pregnancy loss after 20 weeks' completed gestational age), preterm delivery (indicated or spontaneous live birth at < 37 weeks' completed gestational age), delivery of a small for gestational age infant (< 3rd percentile by Oken nomogram) or hypertensive disorder of pregnancy (including preeclampsia without severe features, preeclampsia with severe features, gestational hypertension, superimposed preeclampsia with and without severe features) [15]. Small for gestational age infant was used as it is a reflection of foetal growth potential, thought to be reflective at least in part of the intrauterine environment and maternal exposures [16]. Data were analysed using IBM SPSS and SAS version 9.4 (SAS Institute Inc., Cary, North Carolina) [17].

### 2.3 Statistical Analysis

Data analysis was completed between April 2023 and March 2024. All statistical measures used a 2‐tailed test, and an a priori *p*‐value cutoff of < 0.05 was used to determine statistical significance. Descriptive statistics for sociodemographic and biomedical data included means for continuous variables and frequencies for categorical variables. Logistic models assessed associations between high caffeine intake, APO and individual APOs, adjusted for confounders including age, race, smoking status and educational background, as these have been associated with both caffeine intake and adverse pregnancy outcomes in prior data [18, 19, 20, 21]. As a sensitivity analysis, we examined the association between caffeine in increments of 50 mg/day and increased odds of APO. In a sensitivity analysis, we estimated the *E*‐value, which reflects the strength of unmeasured confounding necessary to shift the odds ratio to 1.20, reflecting a clinically relevant increase in risk [22].

For a secondary outcome of spontaneous miscarriage (foetal loss < 20 weeks), all patients with complete caffeine data and pregnancy outcomes were included. A Cox regression (survival analysis) was used to account for the time‐dependent nature of spontaneous miscarriage. Gestational age was used as the time scale with adjustment for left truncation based on gestational age at study enrolment. Participants were censored at 20 weeks per conventional notation of miscarriage. Caffeine intake was modelled both dichotomously (< 200 mg vs. ≥ 200 mg) and linearly using log‐transformed caffeine intake. Adjusted models included parental age, race (white vs. non‐white), education (high school or less vs. some college or more) and smoking status in the 3 months prior to pregnancy.

## 3 Results

**FIGURE 2:**
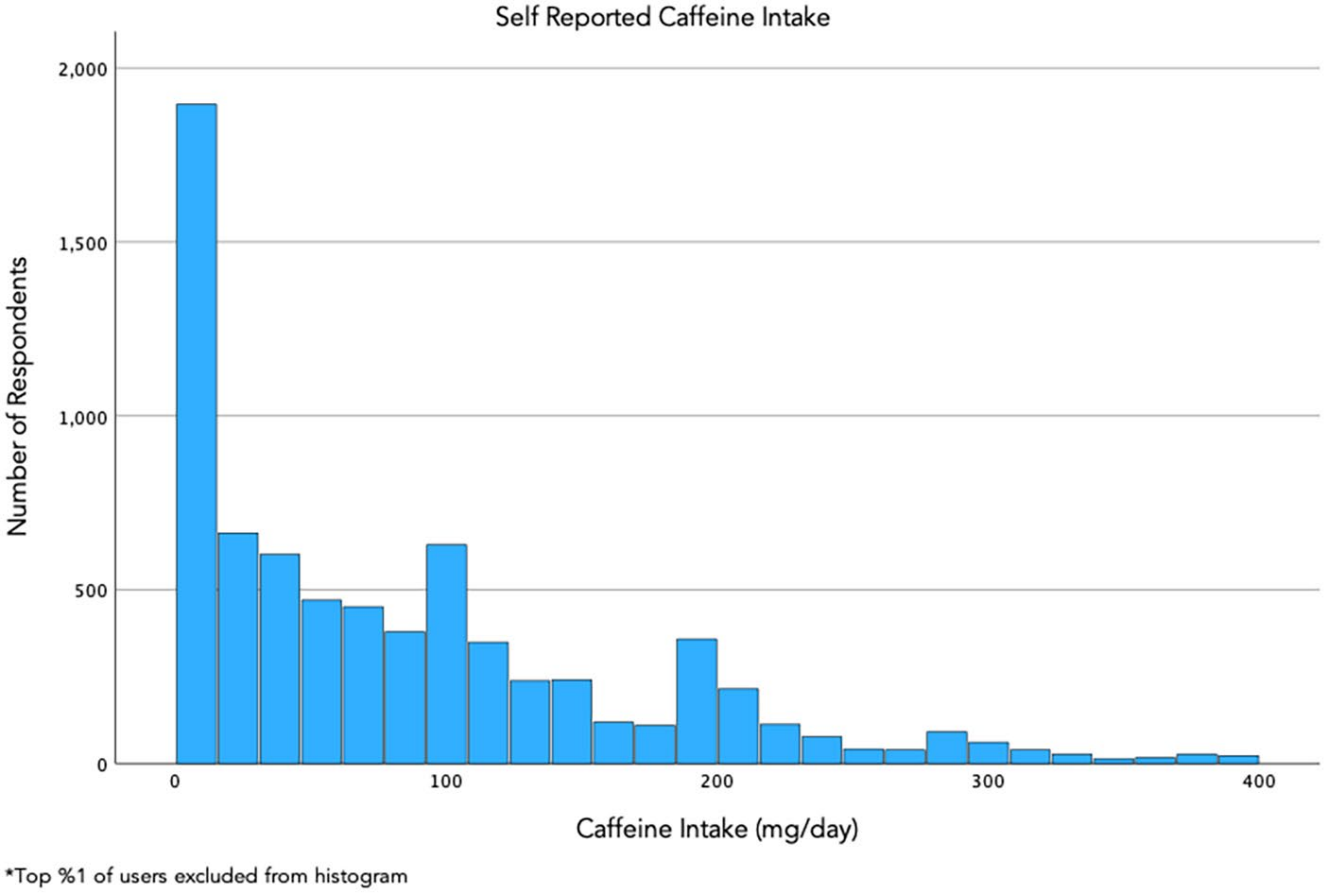
Self‐reported caffeine intake.

7345 patients were included in the final analysis. Median caffeine consumption among all participants was 63.28 mg/day (range 0–739.29, IQR [14.38, 127.57]) (Figure 2). 6504 (88.6%) patients self‐reported low caffeine intake (< 200 mg/day), and 841 (11.4%) patients reported high caffeine intake (≥ 200 mg/day). 2168 (29.5%) patients had an adverse pregnancy outcome, with 1710 (23.3%) of patients experiencing hypertensive disorders of pregnancy, 25 (0.3%) patients having a stillbirth, 147 (2%) with small for gestational age neonates, and 568 (7.8%) patients delivering live preterm infants.

Those who consumed high caffeine levels in the 3 months prior to the first study visit were significantly more likely to be older (29.3 vs. 27.0 years), mean difference −2.27 (95% CI −2.67, −1.88) and to have smoked in the 3 months prior to pregnancy (OR 2.93 [95% CI 2.5–3.44]) (Table 1). There was no significant difference in caffeine consumption by body mass index, insurance status, marital status, and history of chronic hypertension.

**TABLE 1:** Caffeine use and associated demographic and clinical characteristics.

	Overall no (%)	Low caffeine (0 <, < 200) (%)	High caffeine (≥ 200) (%)
Age^a^	27.3 (5.6)	27.0 (5.5)	29.3 (5.5)
BMI in early pregnancy^a^	26.3 (6.3)	26.2 (6.3)	26.6 (6.3)
Race and ethnicity			
Asian	300 (4.1)	276 (4.2)	24 (2.9)
Hispanic	1231 (16.8)	1139 (17.5)	92 (10.9)
Non‐Hispanic Black	805 (11.0)	742 (11.4)	63 (7.5)
Non‐Hispanic White	4661 (63.5)	4039 (62.1)	622 (74.0)
Other	346 (4.7)	306 (4.7)	40 (4.8)
Educational level			
< High school	509 (6.9)	468 (7.2)	41 (4.9)
High school graduate or equivalent	805 (11)	725 (11.2)	80 (9.5)
< College degree	2106 (28.7)	1899 (29.2)	207 (24.6)
≥ Bachelor's degree	3922 (53.4)	3409 (52.4)	513 (61.0)
Insurance status			
Public	1949 (26.7)	1746 (27.0)	203 (24.2)
Private	5353 (73.3)	4718 (73.0)	635 (75.8)
Marital status			
Single	2367 (33.2)	2103 (33.3)	264 (32.4)
Married	4701 (66.0)	4162 (66.0)	539 (66.2)
Other (Divorced, widowed, separated)	54 (0.8)	43 (0.7)	11 (1.4)
Obesity (BMI ≥ 30)	1564 (21.6)	1378 (21.5)	186 (22.3)
Smoke in 3 months prior to pregnancy	1236 (16.8)	954 (14.7)	282 (33.5)
History of chronic hypertension	182 (2.5)	155 (2.4)	27 (3.2)

^a^ Continuous variable results include mean (standard deviation).

For our primary outcome, 260 (30.9%) of high caffeine users had an adverse pregnancy outcome compared to 1908 (29.3%) of low caffeine users (Table 2). After adjustment for confounders, there remained no difference in any adverse pregnancy outcome (aOR 0.99 [95% CI 0.84–1.16]). This finding persisted in a sensitivity analysis using logistic regression, which showed that for every 50 mg increase in caffeine intake, there was no statistically significant increase in odds of APO (aOR 1.01, 95% CI 0.97–1.04, *p* = 0.76). This held true even when caffeine intake was modeled as a categorical variable to allow for greater flexibility (see Table S1). There was similarly no difference in prevalence of each individual adverse pregnancy outcome including stillbirth, hypertensive disorders of pregnancy, preterm birth, and small for gestational age infant by caffeine intake in the periconception period (Table 2). *E*‐values, calculated to represent the minimum strength of unmeasured confounding needed to shift the estimated ORs to 1.2, ranged from 1.6 – 2.6.

**TABLE 2:** Association between caffeine intake and adverse pregnancy outcomes.

	Incidence (%)	OR (95% CI)	*p*	aOR^a^ (95% CI)	*p*	*E*‐value^d^
Any adverse pregnancy outcome						
Low caffeine^b^	1908 (29.3)	1 [Reference]	*0.35*	1 [Reference]	*0.99*	1.7
High caffeine	260 (30.9)	1.08 (0.92–1.26)		0.99 (0.84–1.16)		
Stillbirth						
Low caffeine	21 (0.3)	1 [Reference]	*0.52*	1 [Reference]	*0.49*	NA
High caffeine	4 (0.5)	1.48 (0.51–4.31)		1.49 (0.49–4.58)		
Hypertensive disorders of pregnancy^c^						
Low caffeine	1502 (23.1)	1 [Reference]	*0.29*	1 [Reference]	*0.85*	1.6
High caffeine	208 (24.7)	1.09 (0.93–1.29)		1.02 (0.86–1.21)		
Preterm birth						
Low caffeine	502 (7.7)	1 [Reference]	*0.89*	1 [Reference]	*0.85*	1.9
High caffeine	66 (7.9)	1.02 (0.78–1.33)		0.97 (0.74–1.87)		
Small for gestational age						
Low caffeine	134 (2.1)	1 [Reference]	*0.32*	1 [Reference]	*0.34*	2.6
High caffeine	13 (1.5)	0.75 (0.42–1.33)		0.75 (0.42–1.35)		

Abbreviations: aOR, adjusted odds ratio; CI, confidence interval.

^a^ Model adjusted for age, race, smoking status, and educational background.

^b^ Low caffeine defined as < 200 mg/day; high caffeine defined as 200 mg/day or greater.

^c^ Hypertensive disorders of pregnancy include any diagnosis of: gestational hypertension, preeclampsia (with or without severe features), chronic hypertension with superimposed preeclampsia and eclampsia.

^d^ The *E*‐value reflects the strength of unmeasured confounding necessary to shift the estimated OR to 1.20. No *E*‐value is presented for stillbirth as the estimated OR is greater than 1.20.

There were 35 spontaneous miscarriages in the dataset. In a survival model of miscarriage adjusted for age, race, education, and smoking, the hazard ratio for high caffeine intake (≥ 200 mg/day) was 0.63 (95% CI: 0.19, 2.10). Similarly, when caffeine was explored as a continuous variable, there was no association with spontaneous miscarriage with a 1 standard deviation increase in caffeine intake per day (aHR 0.99, 95% CI 0.69–1.40).

## 4 Discussion

### 4.1 Main Findings

In this analysis we found no statistically significant association between high caffeine consumption in the periconception period and adverse pregnancy outcomes. Further, there was no evidence of an association between self‐reported periconception caffeine intake and subsequent spontaneous miscarriage among this cohort of nulliparous patients.

### 4.2 Strengths and Limitations

Our study has several strengths, most notably in that the cohort represents one of the largest, most diverse prospective groups of American pregnant patients which is uniquely poised to provide data for large‐scale analysis. The prospective nature of this study limits recall bias, which often impacts retrospective caffeine consumption studies, especially among those who had pregnancy loss [13, 23, 24, 25]. The Block Food Frequency Questionnaire has been a validated tool in use for decades, which lends strength to its ability to approximate intake [26]. Additionally, one study which explored caffeine and related biomarker levels in maternal urine and umbilical cord blood found a strong correlation with maternal self‐reported intake, which may offer the benefits of lower cost and greater accessibility [27].

Despite the relative strength of the prospective nature of this study as well as the reliability of the FFQ, limitations of caffeine measurement in this analysis remain. Specifically, misclassification of caffeine intake is a concern as not all caffeine levels are stable among different beverages [13]. Further, as the FFQ was only administered at the first study visit in which participants were asked regarding periconception caffeine consumption, our results may not be entirely reflective of continued consumption during pregnancy. A prior study noted a large majority of patients change caffeine consumption after knowledge of pregnancy, and it is not known if the answers to FFQ reflect continued levels of caffeine consumption during pregnancy [28]. Additionally, there may be non‐differential misclassification of caffeine exposure. Stigma around caffeine exposure in pregnancy may contribute to under‐reporting as well; prior studies which compared self‐reported tobacco use in comparison to biomarkers found a higher nondisclosure rate among pregnant patients than their non pregnant counterparts [29]. Our analysis incorporates covariates such as race, educational background and tobacco use as a proxy for social determinants of health, but we acknowledge there may be other lifestyle factors not incorporated into our analysis that may be confounding. Similarly, the absence of accounting for sleep, which has a known relationship with caffeine consumption and adverse pregnancy outcomes, limits the understanding of these results [30]. To account for unmeasured confounding due to these variables, we additionally present *E*‐values, which range from 1.6 to 2.6, suggesting that moderate unmeasured confounding may explain the null results, though the imprecision of the estimates limits interpretability. Additionally, as the outcomes studied are competing risks for each other (e.g., hypertension and preterm delivery), causal interpretation of individual APOs may be limited. Finally, the lack of uniformity in composite adverse pregnancy outcome variables and potential for bias when combining individual outcomes must be acknowledged, though we also present associations with individual APOs to facilitate interpretation [31].

Lastly, our analysis included miscarriage and stillbirth given the importance of these outcomes in assessing maternal risk and providing counselling. However, the numbers of each that were eligible for analysis were low (*n* = 35 miscarriages, 25 stillbirths), and by entry criteria, patients did have to have a viable pregnancy at the initial visit, which limits our ability to draw significantly meaningful conclusions from the analysis; most miscarriages likely occurred prior to study enrolment. A prior nuMoM2b study that explored sleep position and stillbirth also faced difficulties making conclusions given the relatively small sample size [32]. Thus, larger, targeted studies are needed to confirm our findings on caffeine and lack of association with both miscarriage and stillbirth.

### 4.3 Interpretation

ACOG currently states that caffeine consumption of < 200 mg/day does not appear to be a major contributing factor in miscarriage or preterm birth, and that the relationship to foetal growth restriction is unknown [4]. This assertion is corroborated in part by several other studies. Hinkle's 2021 publication using a large prospective cohort concluded that caffeine consumption is not associated with hypertension risk [9]. Similarly, the Norwegian Generation R Study found no increased risk of preeclampsia with increasing caffeine consumption, and even noted a statistically significant protective effect of caffeine on preeclampsia development among patients who consumed 2–3.9 units per day (approximately 180–350 mg/day, as defined by study authors) [33]. Our findings offer further evidence that self‐reported high periconception caffeine is not associated with these adverse outcomes.

Additionally, our study provides further evidence that caffeine consumption in the periconception period is not associated with the delivery of a small for gestational age neonate. This is consistent with other studies that found no difference between caffeine consumption levels and birth weight, but is in contrast to some, such as the CARE Study Group in the United Kingdom, which found that caffeine consumption during pregnancy was associated with an equivocally increased risk of foetal growth restriction across all levels of caffeine consumption [2, 4, 5, 6]. The Generation R study similarly found an association between caffeine and lower birth weight, but this was only notable when consumption was greater than 6 cups of caffeine per day (equivalent to 540 mg/day per the authors) [33].

In regard to spontaneous miscarriage, our findings are consistent with many prior studies, which found either no impact of caffeine on miscarriage or that its impact could be accounted for by the effect of nausea symptoms [24, 25, 34]. However, Weng et al.'s prospective study of over 1000 pregnant patients did find an increased risk of miscarriage with greater than 200 mg per day of caffeine consumption (aHR 2.23, 95% CI 1.34–3.69) [13]. This may be secondary to patient differences, timing of caffeine consumption report and low N with miscarriage in our study [4, 13]. Ultimately, our sample size and the study's inclusion criteria preclude significant conclusions to be drawn regarding caffeine and spontaneous miscarriage.

## 5 Conclusion

Our analysis suggests that higher levels of caffeine (i.e., above the 200 mg cutoff) reported in the periconception period are not associated with adverse pregnancy outcomes. Prior research has demonstrated that making decisions about nutritional intake in pregnancy is limited by the adequacy of counselling received as well as the pressure to make healthy choices on behalf of the foetus [35, 36]. The data presented may be used to reassure patients that increased periconception caffeine intake does not appear to be associated with adverse pregnancy outcomes, though further robust prospective studies are needed to determine a “safe” cutoff for patients and to better delineate risks of continued consumption throughout pregnancy. Finally, more work is required to determine potential downstream effects of maternal caffeine use on childhood health outcomes [37, 38, 39].

Further, additional work which includes biochemical markers and maternal metabolism is needed to confirm the validity of these results. In fact, caffeine metabolism, mediated by maternal cytochrome P450 1A2 (CYP1A2), has been found to be a reason for caffeine clearance differences as well as adverse pregnancy outcomes [6]. One study found that the association of caffeine intake with FGR was greater among women with faster caffeine clearance than in those with slower clearance (test for interaction, *p* = 0.06) [6]. Individual characteristics may therefore affect the relationship between caffeine and adverse pregnancy outcomes.

This study offers one of the largest population‐based US prospective cohorts exploring the association of self‐reported caffeine intake in the periconception period on adverse pregnancy outcomes. We were able to explore and establish significant differences in caffeine intake by various demographic factors including race, educational level, and age, but found no association with higher caffeine intake and APOs in our adjusted models. As miscarriage and stillbirth were rare in this dataset, conclusions about caffeine's impact on these endpoints cannot be drawn. Our findings may provide patients more comfort and flexibility in the continuation of caffeine consumption in the periconception period. The impact of antenatal caffeine consumption on miscarriage as well as paediatric outcomes is still largely unknown and requires further research prior to modification of current caffeine recommendations.

## Author Contributions

All authors participated in conception and design of the analysis. R.S.R. performed the analysis with supervision from S.C.S. and in consultation with A.A.F. Original draft written by R.S.R. with review and editing from S.C.S. and A.A.F.

## Disclosure

The authors have nothing to report.

## Ethics Statement

NorthShore University Health System Institutional Review Board (EH23‐021) approval was obtained 03/03/2023. A Data Use Agreement between Endeavor Health and the Eunice Kennedy Shiver National Institute of Child Health Human Development Data and Specimen Hub was obtained before data was obtained and analysis began.

## Conflicts of Interest

The authors declare no conflicts of interest.

## Supporting information


**Table S1:** Risk of adverse pregnancy outcome by 50 mg increase in caffeine level.

## Data Availability

The data that support the findings of this study are available from NICHD. Restrictions apply to the availability of these data, which were used under licence for this study. Data are available from https://dash.nichd.nih.gov with the permission of NICHD.

## References

[1] J. Qian , Q. Chen , S. M. Ward , E. Duan , and Y. Zhang , “Impacts of Caffeine During Pregnancy,” Trends in Endocrinology and Metabolism 31, no. 3 (2020): 218–227. 31818639 10.1016/j.tem.2019.11.004 PMC7035149

[2] B. H. Bech , C. Obel , T. B. Henriksen , and J. Olsen , “Effect of Reducing Caffeine Intake on Birth Weight and Length of Gestation: Randomised Controlled Trial,” BMJ 334, no. 7590 (2007): 409. 17259189 10.1136/bmj.39062.520648.BE PMC1804137

[3] Committee RPI , “Healthy Eating and Vitamin Supplements in Pregnancy Royal College of Obstetricians & Gynaecologists,” (2022), https://www.rcog.org.uk/for‐the‐public/browse‐our‐patient‐information/healthy‐eating‐and‐vitamin‐supplements‐in‐pregnancy/.

[4] ACOG Committee Opinion No. 462 , “Moderate Caffeine Consumption During Pregnancy,” Obstetrics and Gynecology 116, no. 2 Pt 1 (2010): 467–468. 20664420 10.1097/AOG.0b013e3181eeb2a1

[5] B. Clausson , F. Granath , A. Ekbom , et al., “Effect of Caffeine Exposure During Pregnancy on Birth Weight and Gestational Age,” American Journal of Epidemiology 155, no. 5 (2002): 429–436. 11867354 10.1093/aje/155.5.429

[6] CARE Study Group , “Maternal Caffeine Intake During Pregnancy and Risk of Fetal Growth Restriction: A Large Prospective Observational Study,” BMJ 337 (2008): a2332. 18981029 10.1136/bmj.a2332 PMC2577203

[7] J. E. James , “Maternal Caffeine Consumption and Pregnancy Outcomes: A Narrative Review With Implications for Advice to Mothers and Mothers‐To‐Be,” BMJ Evidence‐Based Medicine 26, no. 3 (2021): 114–115. 10.1136/bmjebm-2020-111432 PMC8165152 32843532

[8] S. Jahanfar and S. H. Jaafar , “Effects of Restricted Caffeine Intake by Mother on Fetal, Neonatal and Pregnancy Outcomes,” Cochrane Database of Systematic Reviews 2015, no. 6 (2015): CD006965. 26058966 10.1002/14651858.CD006965.pub4 PMC10682844

[9] S. N. Hinkle , J. L. Gleason , S. F. Yisahak , et al., “Assessment of Caffeine Consumption and Maternal Cardiometabolic Pregnancy Complications,” JAMA Network Open 4, no. 11 (2021): e2133401. 34748005 10.1001/jamanetworkopen.2021.33401 PMC8576579

[10] D. M. Haas , C. B. Parker , D. A. Wing , et al., “A Description of the Methods of the Nulliparous Pregnancy Outcomes Study: Monitoring Mothers‐To‐Be (nuMoM2b),” American Journal of Obstetrics and Gynecology 212, no. 4 (2015): 539e1–539e24. 10.1016/j.ajog.2015.01.019 PMC4387083 25648779

[11] J. J. Yland , A. K. Wesselink , T. L. Lash , and M. P. Fox , “Misconceptions About the Direction of Bias From Nondifferential Misclassification,” American Journal of Epidemiology 191, no. 8 (2022): 1485–1495. 35231925 10.1093/aje/kwac035 PMC9989338

[12] L. M. Bodnar , H. N. Simhan , C. B. Parker , et al., “Racial or Ethnic and Socioeconomic Inequalities in Adherence to National Dietary Guidance in a Large Cohort of US Pregnant Women,” Journal of the Academy of Nutrition and Dietetics 117, no. 6 (2017): 867–877. 28320597 10.1016/j.jand.2017.01.016 PMC5446928

[13] X. Weng , R. Odouli , and D. K. Li , “Maternal Caffeine Consumption During Pregnancy and the Risk of Miscarriage: A Prospective Cohort Study,” American Journal of Obstetrics and Gynecology 198, no. 3 (2008): 279e1–279e8. 10.1016/j.ajog.2007.10.803 18221932

[14] E. von Elm , D. G. Altman , M. Egger , et al., “The Strengthening the Reporting of Observational Studies in Epidemiology (STROBE) Statement: Guidelines for Reporting Observational Studies,” Preventive Medicine 45, no. 4 (2007): 247–251. 17950122 10.1016/j.ypmed.2007.08.012

[15] E. Oken , K. P. Kleinman , J. Rich‐Edwards , and M. W. Gillman , “A Nearly Continuous Measure of Birth Weight for Gestational Age Using a United States National Reference,” BMC Pediatrics 3 (2003): 6. 12848901 10.1186/1471-2431-3-6 PMC169185

[16] R. K. Morris , E. Johnstone , C. Lees , V. Morton , G. Smith , and Royal College of O , “Investigation and Care of a Small‐For‐Gestational‐Age Fetus and a Growth Restricted Fetus (Green‐Top Guideline No. 31),” BJOG: An International Journal of Obstetrics & Gynaecology 131, no. 9 (2024): e31–e80. 38740546 10.1111/1471-0528.17814

[17] Corp I , “IBM SPSS Statistics for Macintosh. 29.0 ed. Armonk, NY2022,” (2022).

[18] H. H. Burris and M. R. Hacker , “Birth Outcome Racial Disparities: A Result of Intersecting Social and Environmental Factors,” Seminars in Perinatology 41, no. 6 (2017): 360–366. 28818300 10.1053/j.semperi.2017.07.002 PMC5657505

[19] A. P. Londero , E. Rossetti , C. Pittini , A. Cagnacci , and L. Driul , “Maternal Age and the Risk of Adverse Pregnancy Outcomes: A Retrospective Cohort Study,” BMC Pregnancy and Childbirth 19, no. 1 (2019): 261. 31337350 10.1186/s12884-019-2400-x PMC6651936

[20] B. Tarasi , J. Cornuz , C. Clair , and D. Baud , “Cigarette Smoking During Pregnancy and Adverse Perinatal Outcomes: A Cross‐Sectional Study Over 10 Years,” BMC Public Health 22, no. 1 (2022): 2403. 36544092 10.1186/s12889-022-14881-4 PMC9773571

[21] Ö. Tunçalp , J. Souza , M. Hindin , et al., “Education and Severe Maternal Outcomes in Developing Countries: A Multicountry Cross‐Sectional Survey,” BJOG: An International Journal of Obstetrics & Gynaecology 121, no. s1 (2014): 57–65. 24641536 10.1111/1471-0528.12634

[22] S. Haneuse , T. J. VanderWeele , and D. Arterburn , “Using the E‐Value to Assess the Potential Effect of Unmeasured Confounding in Observational Studies,” JAMA 321, no. 6 (2019): 602–603. 30676631 10.1001/jama.2018.21554

[23] A. T. Hoyt , M. Browne , S. Richardson , P. Romitti , C. Druschel , and National Birth Defects Prevention S , “Maternal Caffeine Consumption and Small for Gestational Age Births: Results From a Population‐Based Case‐Control Study,” Maternal and Child Health Journal 18, no. 6 (2014): 1540–1551. 24288144 10.1007/s10995-013-1397-4 PMC5896301

[24] N. Maconochie , P. Doyle , S. Prior , and R. Simmons , “Risk Factors for First Trimester Miscarriage—Results From a UK‐Population‐Based Case‐Control Study,” BJOG: An International Journal of Obstetrics & Gynaecology 114, no. 2 (2007): 170–186. 17305901 10.1111/j.1471-0528.2006.01193.x

[25] D. A. Savitz , R. L. Chan , A. H. Herring , P. P. Howards , and K. E. Hartmann , “Caffeine and Miscarriage Risk,” Epidemiology 19, no. 1 (2008): 55–62. 18091004 10.1097/EDE.0b013e31815c09b9

[26] B. Boucher , M. Cotterchio , N. Kreiger , V. Nadalin , T. Block , and G. Block , “Validity and Reliability of the Block98 Food‐Frequency Questionnaire in a Sample of Canadian Women,” Public Health Nutrition 9, no. 1 (2006): 84–93. 16480538 10.1079/phn2005763

[27] L. M. Grosso , E. Triche , N. L. Benowitz , and M. B. Bracken , “Prenatal Caffeine Assessment: Fetal and Maternal Biomarkers or Self‐Reported Intake?,” Annals of Epidemiology 18, no. 3 (2008): 172–178. 18083538 10.1016/j.annepidem.2007.11.005 PMC2275917

[28] L. Chen , E. M. Bell , M. L. Browne , C. M. Druschel , P. A. Romitti , and National Birth Defects Prevention S , “Exploring Maternal Patterns of Dietary Caffeine Consumption Before Conception and During Pregnancy,” Maternal and Child Health Journal 18, no. 10 (2014): 2446–2455. 24791972 10.1007/s10995-014-1483-2 PMC5901698

[29] P. M. Dietz , D. Homa , L. J. England , et al., “Estimates of Nondisclosure of Cigarette Smoking Among Pregnant and Nonpregnant Women of Reproductive Age in the United States,” American Journal of Epidemiology 173, no. 3 (2011): 355–359. 21178103 10.1093/aje/kwq381

[30] T. Roehrs and T. Roth , “Caffeine: Sleep and Daytime Sleepiness,” Sleep Medicine Reviews 12, no. 2 (2008): 153–162. 17950009 10.1016/j.smrv.2007.07.004

[31] D. Herman , K. Y. Lor , A. Qadree , D. Horn , and R. D'Souza , “Composite Adverse Outcomes in Obstetric Studies: A Systematic Review,” BMC Pregnancy and Childbirth 21, no. 1 (2021): 107. 33546638 10.1186/s12884-021-03588-w PMC7863533

[32] R. M. Silver , S. Hunter , U. M. Reddy , et al., “Prospective Evaluation of Maternal Sleep Position Through 30 Weeks of Gestation and Adverse Pregnancy Outcomes,” Obstetrics and Gynecology 134, no. 4 (2019): 667–676. 31503146 10.1097/AOG.0000000000003458 PMC6768734

[33] R. Bakker , E. A. Steegers , H. Raat , A. Hofman , and V. W. Jaddoe , “Maternal Caffeine Intake, Blood Pressure, and the Risk of Hypertensive Complications During Pregnancy. The Generation R Study,” American Journal of Hypertension 24, no. 4 (2011): 421–428. 21164492 10.1038/ajh.2010.242

[34] J. L. Mills , L. B. Holmes , J. H. Aarons , et al., “Moderate Caffeine Use and the Risk of Spontaneous Abortion and Intrauterine Growth Retardation,” Journal of the American Medical Association 269, no. 5 (1993): 593–597. 8421363

[35] R. Bagherzadeh , T. Gharibi , B. Safavi , S. Z. Mohammadi , F. Karami , and S. Keshavarz , “Pregnancy; an Opportunity to Return to a Healthy Lifestyle: A Qualitative Study,” BMC Pregnancy and Childbirth 21, no. 1 (2021): 751. 34740317 10.1186/s12884-021-04213-6 PMC8569967

[36] K. Edvardsson , A. Ivarsson , E. Eurenius , et al., “Giving Offspring a Healthy Start: Parents' Experiences of Health Promotion and Lifestyle Change During Pregnancy and Early Parenthood,” BMC Public Health 11 (2011): 936. 22171644 10.1186/1471-2458-11-936 PMC3282831

[37] J. Cheng , H. Su , R. Zhu , et al., “Maternal Coffee Consumption During Pregnancy and Risk of Childhood Acute Leukemia: A Metaanalysis,” American Journal of Obstetrics and Gynecology 210, no. 2 (2014): 151e1–151e10. 10.1016/j.ajog.2013.09.026 24060443

[38] C. Galera , J. Y. Bernard , J. van der Waerden , et al., “Prenatal Caffeine Exposure and Child IQ at Age 5.5 Years: The EDEN Mother‐Child Cohort,” Biological Psychiatry 80, no. 9 (2016): 720–726. 26444074 10.1016/j.biopsych.2015.08.034

[39] J. L. Gleason , R. Sundaram , S. D. Mitro , et al., “Association of Maternal Caffeine Consumption During Pregnancy With Child Growth,” JAMA Network Open 5, no. 10 (2022): e2239609. 36315142 10.1001/jamanetworkopen.2022.39609 PMC9623443

