# Supplementary material for: Periconceptual Caffeine Intake and Adverse Pregnancy Outcomes: Results From the nuMoM2b Cohort
Source: BJOG. 2025 Sep 23;133(2):326–33. doi: 10.1111/1471-0528.70018 (PMC12678039; doi:10.1111/1471-0528.70018)
Supplement: Supplementary file 1 — Table S1: Risk of adverse pregnancy outcome by 50 mg increase in caffeine level. [file BJO-133-326-s001.docx]

Table S1.Risk of adverse pregnancy outcome by 50 mg increase in caffeine level

| Caffeine Level (Referent to 0<50 mg/day) | Adjusted Odds Ratio (aOR) for Adverse Pregnancy Outcome | aOR for Stillbirth | aOR for Hypertensive Disorder of Pregnancy | aOR for Preterm Birth | aOR for Small for Gestational Age |
| --- | --- | --- | --- | --- | --- |
| <50 mg | 1.00 | 1.00 | 1.00 | 1.00 | 1.00 |
| 50 < 100 mg | 1.02 (CI 0.89-1.17) | 0.39 (CI 0.09-1.74) | 1.07 (CI 0.93-1.24) | 1.06 (CI 0.85-1.34) | 0.81 (CI 0.52-1.26) |
| 100 < 150 mg | 1.07 (CI 0.92-1.25) | 1.12 (CI 0.36-3.56) | 1.08 (CI 0.92-1.28) | 1.02 (CI 0.79-1.33) | 0.96 (CI 0.59-1.55) |
| 150 < 200 mg | 1.00 (CI 0.82-1.20) | 1.46 (CI 0.40-5.38) | 1.11 (CI 0.91-1.36) | 0.78 (CI 0.55-1.11) | 0.69 (CI 0.35-1.35) |
| 200 mg + | 1.00 (CI 0.84-1.19) | 1.39 (CI 0.41-4.66) | 1.07 (CI 0.89-1.28) | 0.97 (CI 0.72-1.30) | 0.68 (CI 0.37-1.26) |
